# Selective Early Excision Versus a Conservative Surgical Approach. Management of Deep Burn Injury in a Resource‐Restricted Setting: A Prospective, Observational Study

**DOI:** 10.1002/wjs.70262

**Published:** 2026-02-09

**Authors:** Nikki Allorto, David Gray Bishop

**Affiliations:** ^1^ Pietermaritzburg Metropolitan Department of Surgery University of KwaZulu‐Natal Pietermaritzburg South Africa; ^2^ Perioperative Research Group, Department of Anaesthetics, Critical Care and Pain Management University of KwaZulu‐Natal Pietermaritzburg South Africa

**Keywords:** burn injury, conservative surgical approach, early excision, low‐resource settings

## Abstract

**Background:**

Operative intervention for deep burn injury confers a survival advantage compared to healing by secondary intention and dressings alone. Mortality benefits from early surgery may not extend to resource‐restricted environments. There are major deficiencies in the delivery of advanced burn care in low‐resource settings. This study aimed to compare two different operative approaches in the same setting for deep burn injury.

**Methods:**

We conducted a prospective, observational study at Grey's Hospital in Pietermaritzburg. The first system applied a triage strategy to ensure priority patients received early excision, while the second system provided no excision and performed skin grafting only once spontaneous eschar separation had occurred. The primary outcome was mortality.

**Results:**

The Priority System included 191 admissions with 158 operative admissions. The Conservative System included 199 admissions with 174 operative admissions. The groups were similar in age, total body surface area and sex. Mortality was higher in the Priority versus Conservative System (10.8% vs. 4.6%, *p* = 0.039), with significantly higher acute kidney injury and ICU admission rates but lower sepsis rates. The time from injury to first surgery, and injury to discharge were significantly longer in the Conservative System.

**Conclusion:**

In a single, low‐resource institution, spontaneous eschar separation and delayed skin grafting improved mortality outcomes compared to a triage system providing early excision and subsequent grafting for a group of high‐risk patients. Conservative surgical approaches should be considered where resource restrictions prevent early excision and simultaneous closure. Early excision without immediate closure does not provide mortality benefit.

## Graphical abstract

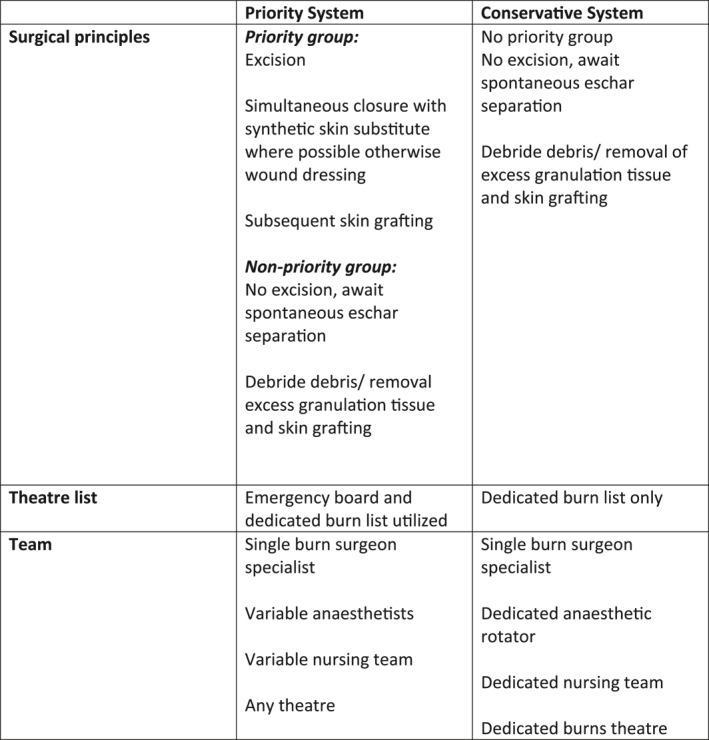

## 1 Introduction

Operative intervention is essential in deep burn injury where burn depth includes deep dermal, full thickness and fourth‐degree burns. In high‐income settings, this consists of excision and skin grafting, with or without temporary coverage or dermal substitution. Excision is defined as removal of the burn eschar before spontaneous separation can occur, where early excision is considered within 10 days of injury, and delayed excision is considered between 10 days and 3 weeks [1]. Current standard of care in high‐income countries is early operative intervention. To achieve superior results by early operative intervention, safe perioperative care must be provided, and closure should be achieved at the time of excision, whether temporary or definitive [2].

There are major deficiencies in the delivery of advanced burn care in low‐resource settings [3]. A systematic review in 2021 showed that in these settings, mortality was higher in early versus late excision, although there was significant heterogeneity in the included papers [4]. This contrasts with the accepted dogma of early excision resulting in lower mortality. It appears that mortality benefits from early surgery may not extend to resource‐restricted environments. However, operative intervention does confer a survival advantage compared to healing by secondary intention and dressings alone [5]. Skin grafting is the definitive intervention and any excision or debridement without eventual skin grafting is of limited benefit, although poorly described in the literature. Provision of skin grafting is available in only 35%–40% of units in low resource‐settings, with major deficiencies in perioperative care [6].

The Pietermaritzburg Burn Service (PBS) provides autologous skin grafting to all deep burn injuries, but capacity for early excision is more challenging due predominantly to limited theater access. Following the implementation of a triage strategy in 2022 to improve access to early excision and grafting for patients deemed to benefit the most, severe system constraints led to further limitation in theater access during 2023. This prevented access to emergency theater lists, restricting surgery to a single operating list per week. In response, a conservative surgical approach was adopted. This approach entailed awaiting spontaneous eschar separation and performing delayed skin grafting. With this method, we still aimed to provide definitive skin grafting in all patients, but without surgical excision. This reduced the operative load to meet resource constraints and provided a unique opportunity to compare operative strategies where provision of early excision and closure is not feasible, and the underlying goal is to graft all deep burns. Research in low‐income settings has not shown early excision to be beneficial, and has focused on early versus late excision, with the critical importance of closure with autologous skin grafting often overlooked.

This study aimed to compare these two different approaches to operative intervention in the same setting for deep burn injury. The first system applied a triage strategy to ensure priority patients received early excision and subsequent grafting, while the second system provided no excision and only skin grafting once spontaneous eschar separation had occurred. The primary outcome was mortality, with secondary outcomes being time from injury to burn service discharge, time form injury to first surgery, infections, acute kidney injury, intensive care admission and graft loss.

## 2 Materials and Methods

We conducted a prospective, observational study at Grey's Hospital in Pietermaritzburg, KwaZulu‐Natal, South Africa. Data collection was part of daily clinical processes and was collected by the principal investigator. Class approval for the Pietermaritzburg Burn Database was approved by the Biomedical Research Ethics Committee at the University of KwaZulu Natal (BCA 106/14). Data were collected and managed using REDCap electronic data capture tools hosted at the University of KwaZulu Natal [7]. Demographic, injury and outcome data collected on all patients admitted to the Greys Hospital Burn Service were used to describe the whole population. Patients who received operative intervention were included in the analysis for primary and secondary outcomes, and compared to patients meeting priority criteria in a previous study where an early surgical approach was implemented (BREC/00004082/2022) over the preceding 18‐month period [8].

### 2.1 Setting

Grey's Hospital is a tertiary hospital in Pietermaritzburg, KwaZulu‐Natal, South Africa. The PBS consists of a regional hospital (specialist support from the tertiary hospital is provided) and a tertiary hospital with an onsite specialist burn surgeon and 12 dedicated burn beds. There is one dedicated burn theater list per week at the tertiary hospital. Patients initially present to other institutions and are referred to Grey's Hospital via Vula, a medical referral application [9], directly to the specialist surgeon. Larger surface area burns are usually transferred immediately, but stable patients will often remain at district or regional level until the need for skin grafting becomes clear, or until development of a complication like infection necessitating a higher level of care.

At Grey's Hospital, in the acute setting, standard resuscitation with balanced crystalloid solutions is performed, and early enteral feeding is commenced for any injury > 15% total body surface area (TBSA). A multidisciplinary team is involved from the time of admission, including occupational therapy, physiotherapy, dietetics and psychology. There are well‐established local protocols for the management of pain and infection based on global guidelines. Patients requiring organ support are referred to the general ICU and compete with other medical and surgical patients for admission. The surgical team consists of a single specialist with 2 rotating junior doctors. The specialist burn surgeon is involved in every operation. After hours, burn patients are looked after by the general surgical team on call. Anesthesia on elective lists is provided by specialists that are experienced with burns anesthesia. They work with a junior anesthetist doing a 6–12 weeks rotation. The junior anesthetist joins the multidisciplinary team ward round weekly and is responsible for the dedicated theater list once a week. For the priority triage system, the emergency board was utilized between 8 a.m. and 10 p.m. Surgery was still performed by the single specialist surgeon, but supported by a variety of junior anesthetists and nursing teams not necessarily familiar with burns, and variable operating theaters depending on availability, that did not necessarily meet the required standards for aspects like temperature control. Tangential excision was performed using a humby knife. Allograft is not available, and when available a temporary synthetic skin substitute was used (Keragentrix MedikaSA, South Africa) This is an acellular bilaminar bovine collagen matrix partially embedded nylon, with outer silicone film. Grafting was performed using an electric dermatome. The systems are summarized in Table 1.

**TABLE 1:** Characteristics of priority system versus conservative system.

	Priority system	Conservative system
Surgical principles	*Priority group*: Excision Simultaneous closure with synthetic skin substitute where possible otherwise wound dressing Subsequent skin grafting *Non‐priority group*: No excision, await spontaneous eschar separation Debride debris/removal excess granulation tissue and skin grafting	No priority group No excision, await spontaneous eschar separation Debride debris/removal of excess granulation tissue and skin grafting
Theater list	Emergency board and dedicated burn list utilized	Dedicated burn list only
Team	Single burn surgeon specialist Variable anesthetists Variable nursing team Any theater	Single burn surgeon specialist Dedicated anesthetic rotator Dedicated nursing team Dedicated burns theater

Inhalation injury was diagnosed using bronchoscopy within 72 h of injury. Acute kidney injury was marked as present if any of the KDIGO (Kidney Disease: Improving Global Outcomes) criteria were present at any time during admission. Infection is a difficult diagnosis to make and was always made by the burn specialist surgeon in conjunction with an intensivist, and not by junior doctors. Infection was always carefully considered, and diagnosis was based on clinical evaluation according to international guidelines, blood and Xray investigation and cultures. Antibiotics were initiated for suspected infection. Sepsis was marked present if systemic antimicrobials were initiated where the cause may have been pneumonia, the wound, line or urinary tract. Graft loss was marked present if more the 5% of the graft surface area failed, as assessed by the single burn specialist.

Wound care follows a specific approach with the use of non‐occlusive, non‐debriding, antimicrobial sheets specifically Cutimed Sorbact (Abigo Medical AB, Askersund, Sweden), after cleaning with Chlorhexidine soapy water. Secondary gauze or Drawtex (Beier Drawtex, South Africa) sheets if exudate was high were placed over the primary dressing. The dressing was changed twice weekly or thrice if needed. No gels, creams or ointments were used.

Due to the lack of burn unit infrastructure, access to isolation, specialized nursing and intensive care and theater access, there are clear institutional guidelines for palliative care [10]. Palliative care was considered for deep injuries above 30%–40% TBSA depending on patient age and mortality scoring.

### 2.2 Statistics

The sample size was based on convenience sampling, over two 18‐month periods. Data analysis was conducted using StataNow SE version 19 (StataCorp, USA). Baseline characteristics of the included patients were reported as mean (standard deviation [SD]) for continuous normally distributed variables; median (interquartile range [IQR]) for data not normally distributed; and count (percent) for categorical variables. For categorical data, we used chi‐squared tests and for non‐parametric continuous data we used the Kruskal–Wallis test. We used the Strengthening the Reporting of Observational Studies in Epidemiology (STROBE) statement guidelines to report our study [11].

## 3 Results

Both systems included 18‐month periods of data collection. The Priority System was applied from January 1, 2022 to June 30, 2023. There were 191 admissions with 158 operative admissions. The Conservative System was applied from 1 July, but with data collection from October 1, 2023 to March 30, 2024. This allowed three months for the clinicians to become familiar with the new surgical approach. During the second phase, there were 199 admissions with 174 operative admissions. The groups were similar in age, TBSA and sex, as illustrated in Table 2. Mechanism of injury in the Priority system was hot water in 44%, flame in 31%, low voltage electrical injury in 8%, high voltage in 5% and the remainder including hot food, hot oil, hot surface, and chemical burns. In the Conservative system, hot water was the mechanism in 48%, flame in 28%, low voltage electrical injury in 4%, high voltage in 5% and the remainder including hot food, hot oil, hot surface, and chemical burns.

**TABLE 2:** Population characteristics of priority and conservative systems.

	Priority system	Conservative system	*p* value
Age	17.7 (19.9)	17.1 (18.9)	0.78
TBSA	13.4 (11.3)	12.7 (9.4)	0.55
TBSA deep	8.9 (9.0)	8.4 (7.8)	0.57
Children < 12 years	87/158 (55%)	102/174 (59%)	0.74
Female	73/158 (46%)	67/174 (39%)	0.16

*Note:* Data are presented as mean (SD) or number (percentage).

Abbreviation: TBSA, total body surface area.

Mortality, acute kidney injury and ICU admission was significantly higher in the Priority System, with lower presence of sepsis. Graft loss was similar in both groups. The time from injury to first surgery, and injury to discharge were significantly longer in the Conservative System (22 [IQR 13–41] vs. 32 [IQR 21–50; *p* < 0.01] days; and 38 [IQR 26–59] vs. 51 [34–72; *p* < 0.01] days respectively). Outcomes between systems are presented in Table 3.

**TABLE 3:** Outcomes between priority and conservative systems.

	Priority system *n* = 158	Conservative system *n* = 174	*p* value
Mortality	17 (10.8%)	8 (4.6%)	0.039
Sepsis	88 (55.7%)	123 (70.6%)	0.01
Acute kidney injury	35 (22.1%)	9 (5.2%)	< 0.01
ICU admission	23 (14.6%)	8 (4.6%)	0.002
Graft loss	54 (34%)	62 (36%)	0.82
Days injury to admission (days)	11 [3–31]	20 [5–41]	0.01
Injury to first surgery (days)	22 [13–41]	32 [21–50]	< 0.01
Injury to discharge (days)	38 [26–59]	51 [34–72]	< 0.01
Surgery to death (days)	10 [4–21]	11 [6–60]	0.33

*Note:* Data are presented as median [interquartile range] or number (percentage).

Abbreviation: ICU, intensive care unit.

## 4 Discussion

This study aimed to compare two different approaches to operative intervention in deep burn injury in a low‐resource setting where access to surgery is limited. In the Priority System, high priority injuries were triaged to receive early excision (which occurred on median day 7 [4–14.5]) using a simple, objective tool, with the non prioritized patients managed by spontaneous eschar separation and delayed grafting (which occurred on median day 33 [21–55]) [8]. Triage strategy is well described in mass casualty literature. We sought to use this concept to implement a simple, reproducible strategy in a setting where our health system was unable to meet the demand. This systematic approach selected patients to be prioritized patients for early surgery based on a more urgent need and greater potential benefit [8]. In the Conservative System, no patients received early surgery, and all were treated conservatively by spontaneous eschar separation and delayed grafting. Importantly, all patients received skin grafting. The main findings were earlier operative intervention and shorter length of stay in the Priority System, but with higher mortality, acute kidney injury and ICU admission. In the Conservative System, operative intervention was 10 days later, with increased length of stay and infection rates, but significantly lower mortality (4.6% vs. 10.8%).

Studies from other low‐income settings on the timing of surgical intervention suggest that the poorest outcomes are seen when there is no surgical intervention for deep burns that is failure to provide autologous skin grafting [5]. If excision is performed, grafting should be done, but when performing excision, early is not better than late [12]. However, there are no studies that compare a conservative approach of eschar separation with delayed skin grafting to any other surgical intervention, as we have done.

Authors from Indonesia have shown the highest mortality when no surgical intervention is provided, with the best outcomes seen when skin grafting was performed at the time of excision, irrespective of timing [5]. The median [IQR] time to operation was 6 [1–28] days. They also describe achieving early excision and grafting in only a small number of patients, with the need for screening and triage to select candidates who would benefit the most. There are similar findings by the group in Malawi assessing operative intervention and mortality in patients who had sustained flame burns. Mortality was significantly lower in the operated versus non‐operated group (3.2% vs. 39.7%). The median [IQR] time to surgery was 18 [9–38] days, with 40.9% having excision only and 53.3% having excision and grafting. They highlight that excision without skin grafting had no mortality benefit [13]. Earlier work by the same group when comparing early versus late excision shows mortality of 25.3% versus 9.2% respectively, where early surgery was performed on median [IQR] day 2 [1–4] and late on day 14 [9–25]. The late excision group showed longer length of stay and higher incidence of fever [14].

Surgeons in low‐resource settings have attempted to achieve early excision for deep burns due to the outcome benefits described for this approach in high‐resource settings [5, 8, 12, 13]. The high burden of resource‐to‐injury ratio has made this challenging. However, the focus has been excision alone, and not excision and skin closure. Even in our own service when prioritizing patients for early surgery we were only able to provide closure at the time of excision in 30% of cases. This is similar to other authors [5, 13]. It is becoming clearer that there are challenges with provision of skin closure [12, 14] and the lack of skin closure is likely to be driving the poorer outcomes. Provision of skin closure may be limited by access to temporary substitutes like allograft or synthetic options [15, 16]. Other contributory factors may include the need to limit operative time, and to stage surgery in order to minimize the perioperative physiological insult. However, utilization of limited resources (theater access, surgical skill, perioperative care) to perform excision without skin closure does not provide benefit. The focus of operative intervention in low‐resource settings should be of skin closure by autologous skin grafting, particularly when access to the operating theater and perioperative care is limited.

The physiological derangement in the burn injured patient is driven by both the burn eschar and the lack of skin integrity. Unless removal of the eschar and closure of the skin deficit has been achieved, there is failure to achieve physiological source control and the critical illness of the burn patient continues. Essential in the management of these patients is the perioperative care, which begins from the time of injury and continues for a protracted period. It includes fluid and electrolyte management, nutritional support, management of pain and infection, transfusion, organ support, occupational therapy, physiotherapy, and psychosocial care. Providing successful surgical intervention requires organization, planning, and communication; in conjunction with the other pillars of burn care [17]. In the operating theater, temperature control, patient positioning, speed of surgery, and number of surgeons involved in major surgery may all influence patient outcome. It is not sufficient to merely provide an operation. Most surgeons understand that non‐viable tissue should be removed, and there is increasing awareness of the global aim for early excision. However the critical importance of skin grafting is undervalued and is not the focus of the general surgeon treating burns in South Africa. In a local study exploring knowledge and access to resources, only 62% of surgeons in training understood the need for skin grafting in full thickness burn injuries [18]. It is imperative for low‐resource settings to understand that the definitive goal in management of deep burn injury is resolution of the wound. This is only achieved by autologous skin grafting. In settings where access to operative intervention is limited, the focus should be on provision of skin grafting. Literature from low‐resource settings exploring operative intervention describe the poorest outcomes in patients not receiving skin grafting, yet access to skin grafting is not universal.

In our setting, skin grafting was always performed for deep burn injury. Our comparison focused on expedited excision for a high‐risk group and subsequent grafting, with only 30% receiving closure at the time of excision, versus no excision and utilization of spontaneous eschar separation with a specific wound care strategy and then skin grafting. The requirement for increased access to the operating theater necessitates utilization of the emergency board, with variable teams and theaters. We believe the difference in outcomes of the two groups can be explained in part by the differences in perioperative care. The utilization of anesthetic teams not familiar with burn surgery and operating in theaters not always temperature controlled is challenging. While we were able to provide earlier surgery for some patients, the outcome benefits of early surgery were potentially offset by the altered perioperative care. Improving perioperative care should be the focus of future quality improvement projects. However, the failure to provide closure at the time of excision in 70% of patients, may be the primary driver of poor outcomes in the Priority System. Our Priority System focused on early excision in selected patients. When the health system constraints limit surgical intervention, the priority should be on provision of autologous skin grafting. If early excision is performed in the absence of skin closure, outcomes are worsened, as seen in our study.

### 4.1 Strengths and Weaknesses

Due to the burn service being managed by a single specialist, there was consistent application of the system and a single surgeon providing operative intervention with reduced inter‐individual bias. Our study did not include data on long term outcomes, such as hypertrophic scarring and contracture, which might be expected in patients with delays from time of injury to surgery. The lack of data on the long‐term outcomes of scarring and function is an important limitation. Data were not collected on all components of perioperative care and this should be the focus of future research as a means toward improving outcomes when operative intervention is provided.

## 5 Conclusion

In a single, low‐resource institution, spontaneous eschar separation and delayed skin grafting improved mortality outcomes compared to a triage system providing early excision and subsequent grafting for a group of high‐risk patients. When early surgical intervention is provided, perioperative care is an essential component. Conservative surgical approaches should be considered where resource restrictions prevent simultaneous early excision and closure. Autologous skin grafting is the definitive procedure for deep burn injury and theater time should be used for definitive procedures when in limited supply. Early excision without immediate closure does not provide mortality benefit and in fact worsens outcomes and should not be done.

## Author Contributions

**Nikki Allorto:** conceptualization, investigation, writing – original draft, writing – review and editing, data curation, visualization, formal analysis. **David Gray Bishop:** formal analysis, methodology, supervision, visualization, writing – review and editing.

## Funding

The authors have nothing to report.

## Conflicts of Interest

The authors declare no conflicts of interest.

## Data Availability

The data that support the findings of this study are available on request from the corresponding author. The data are not publicly available due to privacy or ethical restrictions.
